# A randomized controlled trial on the effect of cranial electrotherapy stimulation on depression, anxiety, and craving in addicted male patients undergoing methadone maintenance treatment

**DOI:** 10.1186/s12888-024-06137-9

**Published:** 2024-11-05

**Authors:** Homa Baghaei Ravari, Ali Kheradmand, Mahdi Ghorbani, Alireza Shamsi, Mehdi Khosravi

**Affiliations:** 1 Department of Psychiatry, Taleghani Hospital, Shahid Beheshti University of Medical Sciences, Tehran, Iran; 2 Department of Psychiatry, Taleghani Hospital Clinical Research Development Unit, School of Medicine, Shahid Beheshti University of Medical Sciences, Tehran, Iran; 3 Biomedical Engineering and Medical Physics Department, School of Medicine, Shahid Beheshti University of Medical Sciences, Tehran, Iran; 4 Department of Medical Physics and Engineering, School of Medicine, Shiraz University of Medical Sciences, Shiraz, Iran

**Keywords:** Cranial electrotherapy stimulation, Depression, Anxiety, Drug addiction, Methadone maintenance treatment

## Abstract

**Background:**

Addicted patients undergoing methadone maintenance treatment are prone to several complications and the risk of relapse.

**Objective:**

The present study aims to investigate the effect of cranial electrotherapy stimulation on depression, anxiety, and craving in addicted male people undergoing methadone maintenance treatment.

**Methods:**

This randomized controlled trial study was conducted on 60 male patients referred to Persia addiction treatment center between 2021 and 2022. Patients were randomly divided into two equal treatment and placebo groups. The treatment group received cranial electrotherapy stimulation intervention for 48 sessions of 30 min. Depression and anxiety were evaluated using the Hamilton questionnaire before and after the intervention, and the level of craving was also evaluated with the Federdi 2008 questionnaire.

**Results:**

Comparing the level of depression and anxiety before and after the intervention in both treatment and placebo groups did not show any significant difference (*p* < 0.05). Craving after the intervention was significantly different in both groups and was lower in the treatment group compared to the placebo group (33.43 versus 42.17, *p* = 0.004). In the placebo group, the level of anxiety and depression, and in the treatment group, the level of depression, anxiety and craving for consumption decreased significantly after the intervention compared to before the intervention (*p* < 0.05).

**Conclusion:**

Cranial electrotherapy stimulation did not have a significant effect on reducing the level of depression and anxiety of patients, but it is effective in the reduction of craving in addicted people undergoing methadone maintenance treatment.

**Trial registration:**

This randomized clinical trial was registered on 2022/5/13 with clinical trial code of IRCT20210523051367N1.

**Supplementary Information:**

The online version contains supplementary material available at 10.1186/s12888-024-06137-9.

## Introduction

Addiction is a complex disease that manifests itself with the compulsive use of substances despite its harmful consequences [1]. Opioid use disorder is a widespread health challenge that results in widespread morbidity and mortality worldwide [2]. The annual report of the World Health Organization (WHO) estimates that there are about 200 million opioid addicts in the world (0.6–0.8% of the general population) [3]. Currently, methadone maintenance treatment is recommended as the treatment of choice for opioid use disorder. Previous research studies have reported conflicting results regarding the effect of methadone maintenance treatment on the mental health of addicts. Methadone was reported as the cause of death in 53.5% of substance-related deaths referred to the forensic organization [4].

Nearly 90% of opioid addicts have some type of mental disorders, including depression, antisocial personality disorder, and anxiety [5]. Anxiety and mood disorders associated with addiction can weaken the response to addiction treatment and lead to the return of addiction [6, 7]. Studies have shown that 20 -90% of addicts who are treated will relapse within the next 6 months [8]. Craving can be defined as a strong and resistant desire to consume drugs, which if this desire for drugs is not satisfied, leads to psychological and physical sufferings such as weakness, anorexia, anxiety, insomnia, aggression and depression [9]. Therefore, addiction treatment usually targets craving to reduce the rate of relapse.

Another treatment method for addicted people is the stimulation of specific areas of the brain, which is performed in four main ways: deep brain stimulation (DBS), transcranial magnetic stimulation (TMS), transcranial direct current stimulation (TDCS) and cranial electrotherapy stimulation (CES) [10]. CES is a neuromodulation technique in which constant or alternating currents of low voltage are applied to the human brain through scalp electrodes.

Previous studies on the drug addiction field show that TDCS can be considered as a complementary treatment method along with other common interventions in the field of addiction to nicotine, marijuana, methamphetamine, and crack [11–13]. The main idea of CES is that the application of weak currents can interact with neural processing, modify plasticity and modify behavior by changing brain networks. This technique is now widely used in basic, sports, military and recreational research [14].

Despite recent advances in understanding the neuroscience of addiction, expectations for drug treatments are less than desirable. CES represents a non-pharmacological tool and a testable opportunity for control of anxiety and depression and drug craving [7]. Previous studies didn’t evaluate the effect of CES on drug craving of patients with methadone maintenance treatment. Therefore, in the present randomized controlled trial the effect of CES on depression, anxiety and craving of the addicted people treated with methadone maintenance is investigated.

## Methods

### Participants and setting

This study investigated the effect of CES on depression, anxiety and craving of people undergoing methadone maintenance treatment between the years 2021 and 2022 male people with a history of drug use were being treated with methadone and referred to Persia drug addiction center (Iran, Tehran). The patients were selected randomly. This study has been approved by the ethics committee of Shahid Beheshti University of Medical Sciences with ethical code of IR.SBMU.MSP.REC.1400.043. This randomized clinical trial was registered on 2022/5/13 with clinical trial code of IRCT20210523051367N1. More details on the clinical trial registration are available at: https://fa.irct.ir/trial/57513.

**Fig. 1:**
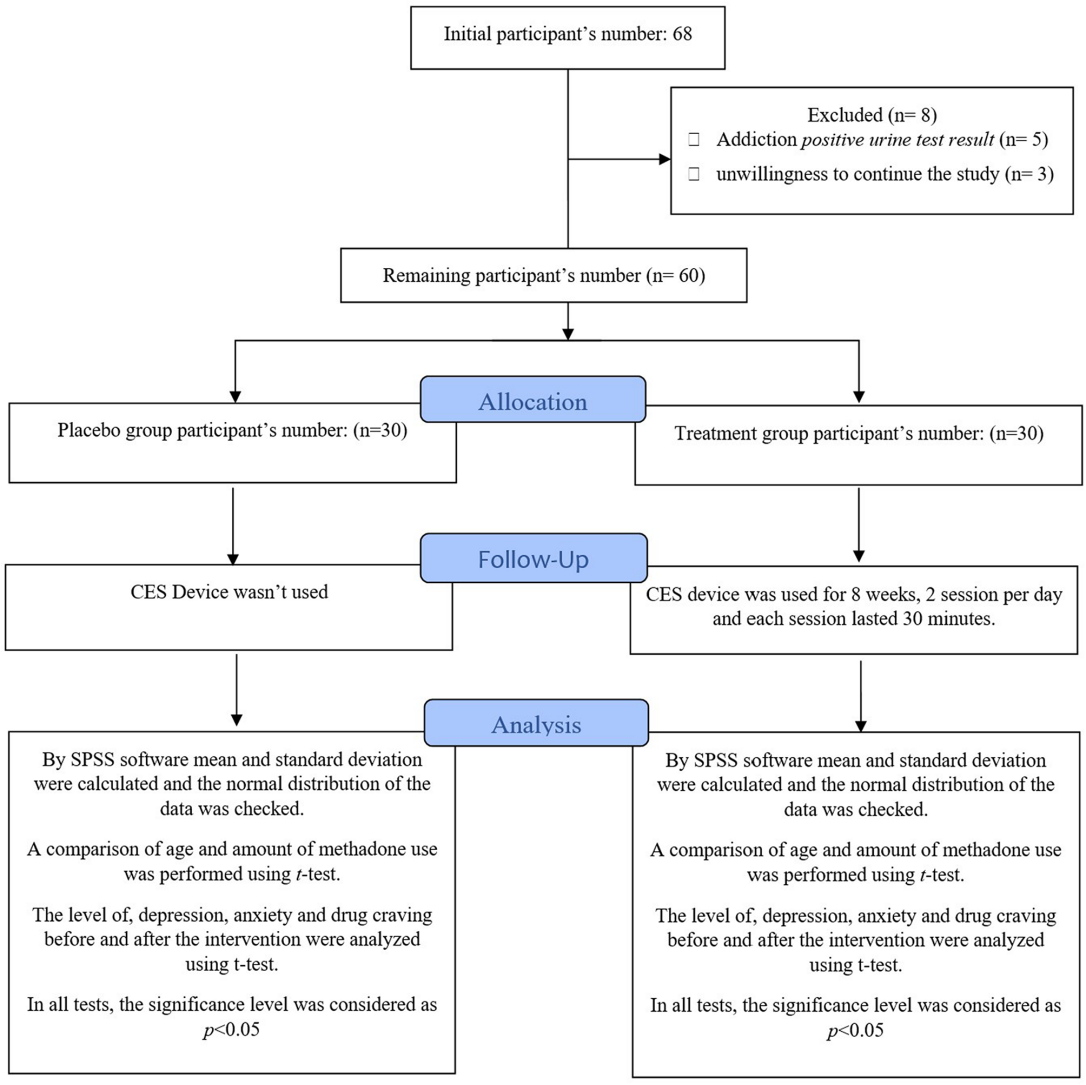
Process flow diagram of the study participants and different steps in this study

The conditions for entering the study included: the first month of starting treatment with methadone, the person’s willingness to participate in the study, not having a serious psychiatric problem and not taking psychiatric medication, and not having a history of head trauma or seizures. Additionally, the people whose urine test result was positive during the study and the people who did not tend to continue participating in the study were excluded from the study. Among all patients who referred to the center, only 68 people were willing to participate in the study. 5 patients due to their positive urine drug testing (UDT) results and 3 patients due to their unwillingness to continue the being studied were excluded. Process flow diagram of the study participants and different steps in this study is illustrated in Fig. 1. Finally, the remaining 60 people were identified and selected by a psychiatric specialist. UDT was taken from each patient before intervention and at the end of the study [15]. The participants were randomly divided into two groups by a psychiatrist with four years of experience, each group included 30 people as placebo and treatment groups. In order to randomize the study, another psychiatrist with ten years of experience divided the participants into groups by block randomization in such a way that the people who were placed in both groups were almost similar in terms of age, gender, the amount of methadone consumed, and the duration of methadone consumption and the people be homogeneous in the two groups. Both patients and the researcher were not aware of the type of treatment. In the placebo group, the device was turned off and in the treatment group, the device was turned on. A nurse with almost twenty years of experience used the device on the patients. The tester did not know whether the device was on or off. In other words, sampling was performed by a two-way blind random method.

### Study intervention

After obtaining the consent form from the test candidates, the level of depression and anxiety was checked using Hamilton’s anxiety and depression questionnaire, The 21-question version of the Hamilton Depression Inventory includes 17 questions about depressive symptoms and 4 questions about other factors and disorders that may be associated with depression. All the questions had scores ranging between 0 and 4 and finally the sum of the scores determined the severity of depression in the patient. After calculating the total scores of the patients, the interviewer started interpreting and determined the status of the patients, depending the total score and the following 4 statuses. Scores 0 to 7 are normal (absence of depression in patient), scores 8 to 16 are mild depression, scores 17 to 23 are moderate depression and 4 scores above 24 are depression [8]. The level of temptation to turn to drugs caving was evaluated by Federdi, Bararfan and Ziyai; 2008 questionnaire, after ensuring the presence of and depression and anxiety at the level of drug craving, an explanation was provided about how to use the CES device.

The weak current applied to the scalp by the CES device had no side effects and the patients were not aware of the stimulation. Since the electrical current is applied to the scalp, as well as the brain, there is usually some local sensory nerve or muscle activation which is easily perceived by the subjects. Also, some Adverse events like headache and skin burn were considered to exclude patients from the study but no symptoms were seen in any of patients. Furthermore, compared to other non-invasive techniques such as transcranial magnetic stimulation and ultrasound stimulation, CES has other advantages, including low cost. Transcranial magnetic stimulation systems are heavy (several kilograms) and large, whereas CES devices are light (less than 1 kg) and small, battery-driven, and therefore can be easily transported, and their potential application at home, allows for increased human trials [16, 17].

**Fig. 2:**
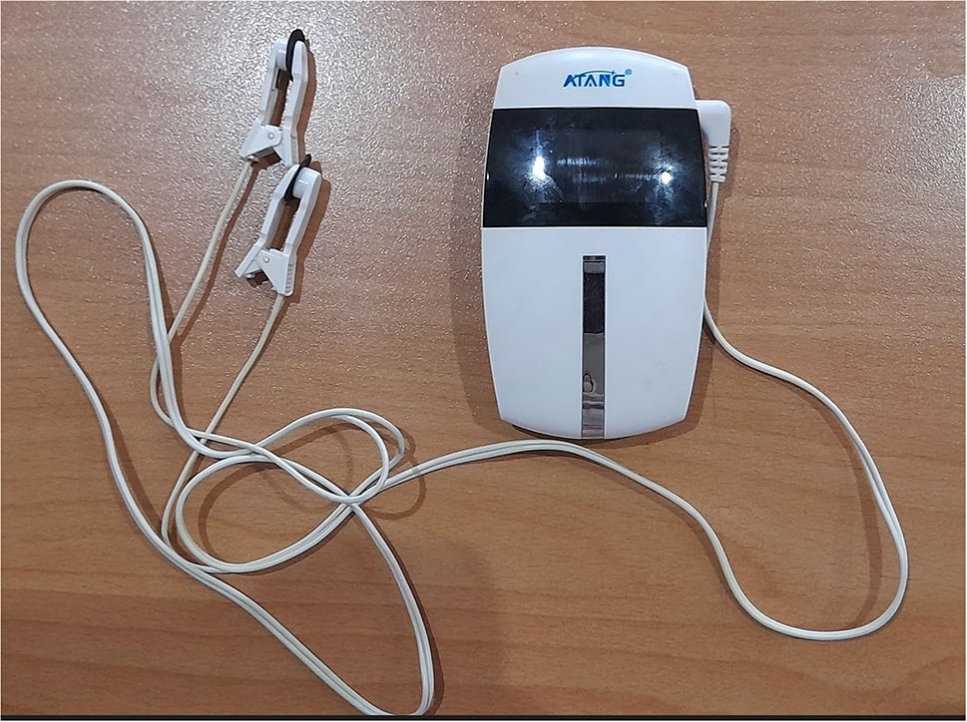
CES device used in this study (ATANG (model AT-9))

**Fig. 3:**
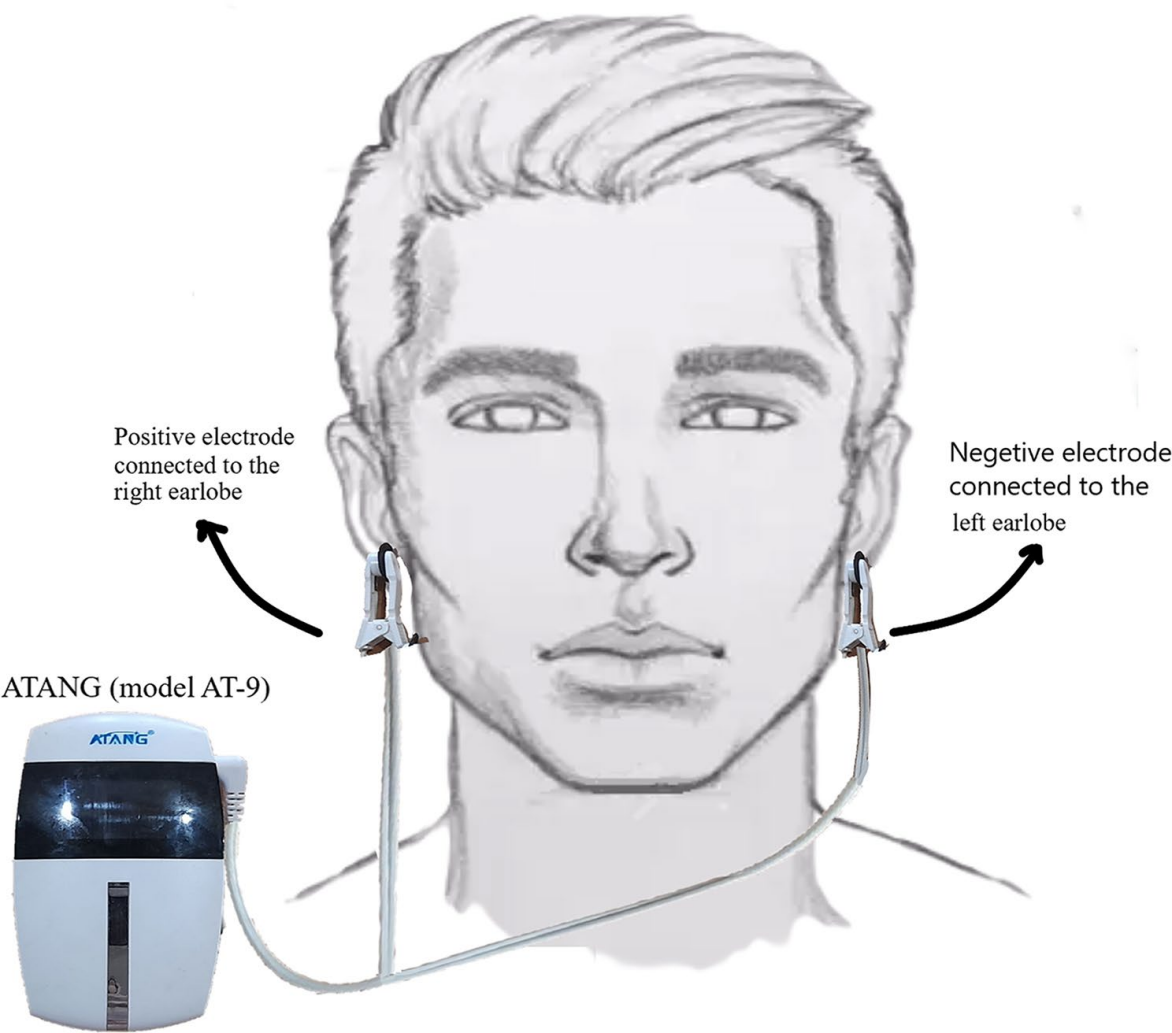
The methods for using a CES device, showing the earlobe clips connections.

This study used an ATANG (ATANG, China, model AT-9) low-frequency CES electrical brain stimulation device (Fig. 2). This device (which has CE standard and FDA approval), is used for treatment of symptoms of depression and anxiety, including decreased energy and fatigue, excessive sleepiness or insomnia, constant sadness, feelings of hopelessness, feelings of worthlessness or helplessness, loss of interest in activities and hobbies, restlessness, and irritability. Chronic, unproven anxiety refers to recurrent and random panic attacks that are commonly associated with major depressive disorder, bipolar disorder, persistent depressive disorder, and generalized anxiety disorder [8, 9]. For stimulation, the electrodes were connected to the earlobes. The methods for using the CES device, showing the earlobe clips connections, are presented in Fig. 3. The device utility was the same for both groups but the device was off for the placebo group. Table 1 shows the specifications of the device. A pulsed electric current with ms pulse duration was passed through two electrodes. Previous studies (such as that by Monte-Silva, et al.) compared different time durations in application of tDCS device. Their results showed that: (1) prolongation of cathodal tDCS duration is able to significantly prolong the aftereffects beyond 1-h duration and; (2) for fractionated usage of tDCS, the interstimulation break is of critical importance. Increasing the stimulation time from 9 to 18 min significantly lengthened the after effects of cathodal tDCS from 60 to 90 min. When the second tDCS session was applied during aftereffects of the first session, inhibitory plasticity was enhanced and prolonged for ≤ 120 min after tDCS application compared with 9-min tDCS. On the other hand, when the second stimulation was performed 3–24 h after the first one, tDCS had a more mixed effect on cortical excitability [18]. In this study, the time and repetition of the CES device usage was influenced by different conditions such as the cooperation of patients and the time of patients’ visits for their initial treatment, as well as the general tolerance of the patients toward the side effects. Therefore, according to experiences from previous studies in this field, the present researchers determined the time and repetition for application of the CES device. The CES device was used for eight weeks, three days a week for two sessions a day, and each session lasted for 30 min. After eight weeks, the depression and anxiety test and the drug craving were scored again in the same way as before the device use. This device has a constant intensity level and also only has a frequency output of 250 Hz.

**Table 1:** Specifications of the ATANG (model AT-9) CES

Impulse range	0–15 Hz
Pulse duration	100 ± 10 ms
Single pulse amplitude	2 ± 0.4 ms
Pulse repetition rate	250 ± 50 Hz
The amplitude value of the output waveform	17 V
Battery voltage	100–240 V
Treatment time	15, 30, 45, 60 min
The temperature of the device working environment	5–40 ºC
Relative humidity	≥ 80%
Atmospheric pressure	86–100 kPa

To perform the study, it was approved by receiving the ethics code for research from Shahid Beheshti University of Medical Sciences, (IR.SBMU.MSP.REC.1400.207). The patient’s dignity and rights as respected, as all the patients had right to quit the study whenever they want. at the end of the research, every person who entered the study as a participant had the right to be informed about the results of this study.

### Statistical analyses

Data were analyzed using Statistical Package for the Social Sciences (SPSS) software (USA, version 22). Data description was performed using mean and standard deviation. The normal distribution of the data was checked and confirmed using the Kolmogorov-Smirnov test and the Normal P-P diagram. A comparison of age and amount of methadone use was performed using *t*-test. The level of, depression, anxiety and craving before and after the intervention in two groups and in each group, as well as the changes in their levels in the two groups, were checked by *t*-test. In all tests, the significance level was considered as *p <* 0.05.

The timeline is as follows. The total of these is about 1 year, patient registration took 1 month, allocation of patients to two groups took 1 week, intervention on people took 4 months, and analysis took 6 months.

## Results

Description of age and methadone consumption in the placebo and treatment groups are presented in Table 2. The results show that the patients in the both placebo and treatment groups were different in terms of age (36.47 years and 39.03 years, respectively, *p* = 0.43).

**Table 2:** Description of age and methadone consumption in the placebo and treatment groups (*t*-test statistical analysis test was used)

Variable	Placebo group = 30 *n*		Treatment group = 30 *n*		*p*-value
	Mean	Standard deviation	Mean	Standard deviation	
Age (year)	36.47	13.35	39.03	11.71	0.43
Amount of methadone consumption (mg)	29.00	7.70	29.50	8.34	0.81

**Fig. 4:**
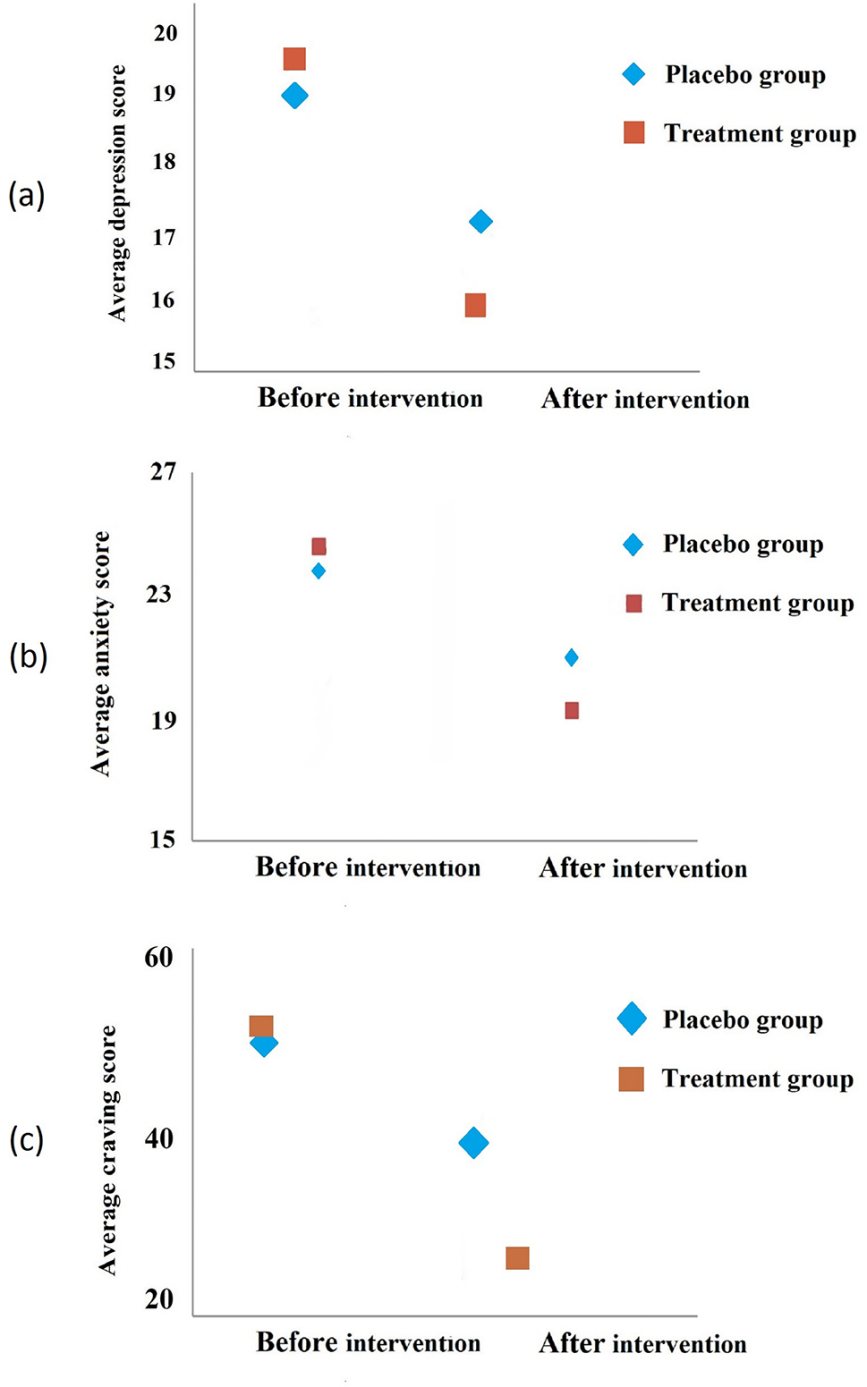
The level of depression (**a**), anxiety (**b**), and craving* (**c**) before and after intervention in the placebo and treatment groups. (*p-value = <0.05)

Average level of depression, anxiety and drug craving scores before and after the intervention in the placebo and treatment groups are presented in Table 3; Fig. 4, the *p-value* was respectively 0.68, 0.43 and 0.53 for before the intervention and 0.26, 0.12 and < 0.01 for after the intervention. The same variables in each group are presented in Table 4, for placebo group the *p-value* for depression was 0.09 and for anxiety and craving was > 0.01 also for treatment group in all three variables the *p-value* was > 0.01. Changes (percent) in depression, anxiety and drug craving scores before and after 8 weeks of the intervention in the placebo and treatment groups are listed in Table 5, the *p-value* was respectively 0.02, 0.01 and > 0.01 for depression, anxiety and drug craving.

**Table 3:** Average level of, depression and anxiety drug craving scores before and after the intervention in the placebo and treatment groups (*t*-test statistical analysis test was used)

	Variable	Placebo group = 30 *n*		Treatment group = 30 *n*		*p*-value
		Mean	Standard deviation	Mean	Standard deviation	
Before the intervention	Depression	18.93	4.82	19.43	4.67	0.68
	Anxiety	23.77	3.99	24.60	4.14	0.43
	Craving	51.40	12.05	53.33	11.24	0.53
After the intervention	Depression	17.00	3.87	15.70	4.86	0.26
	Anxiety	20.93	3.63	19.27	4.35	0.12
	Craving	42.17	11.39	33.43	11.31	0.01>

**Table 4:** Average level of depression, anxiety and drug craving scores before and after the intervention in each group(*t*-test statistical analysis test was used)

	Variable	Before the intervention		After the intervention		*p*-value
		Mean	Standard deviation	Mean	Standard deviation	
Placebo group = 30 *n*	Depression	18.93	4.82	17.00	3.87	0.09
	Anxiety	23.77	3.99	20.93	3.63	0.01>
	Craving	51.40	12.05	42.17	11.39	0.01>
Treatment group = 30 *n*	Depression	19.42	4.67	15.70	4.86	0.01>
	Anxiety	24.60	4.14	19.27	4.35	0.01>
	Craving	53.33	11.24	33.43	11.31	0.01>

**Table 5:** Changes (percent) in depression, anxiety and drug craving scores before and after 8 weeks of the intervention in the placebo and treatment groups (*t*-test statistical analysis was used)

Variable	Placebo group = 30 *n*	Treatment group = 30 *n*	*p*-value
Depression	-8.27	-18.76	0.02
Anxiety	-11.10	-21.22	0.01
Craving	-17.61	-37.42	0.01>

In Table 6 comparison between methadone consumption and craving in the treated group is performed.

**Table 6:** Comparison between methadone consumption and craving in the treated group(*t*-test statistical analysis test was used)

Evaluation time	Amount of methadone consumption (mg)				*p*-value
	30<		≤ 30		
	Mean	Standard deviation	Mean	Standard deviation	
Before intervention = 30 n	53.92	8.19	52.94	13.10	0.82
After intervention = 30 n	31.50	10.73	31.72	11.81	0.46

The results of Table 6 show that there is a significant difference in the amount of methadone consumption and craving in the treated group (*p* < 0.05). The amount of methadone consumption (29.00 mg and 29.50 mg, respectively, *p* = 0.81) had no significant difference and was homogeneous see (Fig. 4).

## Discussion

Drug addiction is one of the most serious and global public health problems. According to a recent survey, different addictions cause the death of 11.8 million people per year worldwide [19]. Several treatment methods have been developed for psychoactive substance use disorders [20]. Although maintenance treatment is provided in reducing drug craving, relapses and physical complaints of opioid users after withdrawal, some patients still experience abstinence during the first years [21].

Regarding to Table 2, the ages of the patients in the placebo and treatment groups are not significantly different (*p* = 0.43). Based on the obtained results (Fig. 4), there was no significant difference between the variables of depression, anxiety and craving in the placebo and intervention groups before the intervention (*p = >* 0.05). Additionally, After the intervention, the level of depression and anxiety in the two groups was not significantly different (*p = >* 0.05), while the average drug craving in the intervention group (33.43) was significantly lower than the placebo group (42.17) (*p* = < 0.01).

Examining the level of depression, anxiety and drug craving for drug consumption separately in each group, before and after the intervention, showed that after the intervention in the placebo group, except for depression (*p* = 0.09), the level of anxiety and drug craving decreased (for both *p* = < 0.01), and in the treatment group, all the investigated variables decreased significantly (*p* = < 0.01 for all three variables) (Table 4; Fig. 4). Examining the rate of reduction showed that in the treatment group that was affected by CES, compared to the placebo group, the percentage of changes in anxiety (-21.20% versus − 11.10%), depression (-18.80% versus − 8.30%) and craving (-37.40% versus − 17.60%) was significantly higher after eight weeks of intervention (Table 5).

In general, the results of our study show that the CES device was not effective in reducing the depression and anxiety of the participants (because the level of depression and anxiety decreased in both groups), however, the use of CES could reduce the drug craving. Because there was a significant difference in drug craving after the intervention in the two groups, and the rate of reduction in drug craving in the treatment group was higher than that in the placebo group (-37.42 versus − 17.61) (Table 5).

CES is a neuromodulation technique that delivers low-intensity pulsed current to cortical areas and facilitates or inhibits spontaneous neural activity. In the past 10 years, the physiological mechanisms of action of CES have been intensively investigated to support the investigation of its applications in clinical neuropsychiatry and rehabilitation. This technique is simple, safe and cheap and has a very portable device [22, 23].

Recent neuroimaging studies have identified cortical areas associated with drug craving. Most of these studies show that the prefrontal areas of the brain, especially the dorsolateral prefrontal cortex, play a major role in craving for drugs and smoking [24, 25]. Furthermore, the use of transcranial magnetic stimulation, a technique that can transiently modulate focal cortical activity, has shown that high-frequency transcranial magnetic stimulation in the dorsolateral prefrontal cortex leads to significant reductions in smoking and cocaine craving [26, 27]. In studies of CES with the aim of stimulating the dorsolateral prefrontal cortex, it was shown that modulating this area is accompanied by behavioral changes such as cognitive changes in healthy people [28] and patients with depression and mood changes in depressed patients [29]. Previous studies that examined neural responses to cues of craving in nicotine users showed that the anterior cingulate, amygdala, insula, dorsolateral frontal cortex, and orbitofrontal cortex were associated with craving [30] so, probably simulating by CES can impact this brain part.

The cause and pathogenesis of drug addiction is related to the change in the function of multiple brain systems. These include altered glutamate, opioid, cannabinoid, gamma-aminobutyric acid, norepinephrine, and the serotonergic system. In addition, the mesolimbic dopaminergic reward system plays the main role in the pathogenesis of addiction, and the low functioning of this system is one of the key features of drug addiction [31]. Chronic use of addictive substances leads to increased dopaminergic reward pathway activity [32]. In addition, drug abstinence is associated with decreased activity of the dopaminergic reward pathway, which in turn activates craving and relapse [33]. Human and animal model studies have shown that stimulation of the frontal cortex leads to the release of dopamine in the mesolimbic pathway. Increased stimulation in the dopaminergic pathway may act like the substance’s effect in the mesolimbic pathway, leading to a temporary reduction in craving, The cortex is the area that regulates attention and motor output [34]. Another possibility is that phasic dopamine release promotes drug-seeking behavior and motivates individuals to focus on stimuli and approach goal-directed behavior [35]. Stimulation of the dorsolateral prefrontal cortex by CES may increase the phasic release of dopamine, thereby reducing the desire to use substances [36].

Similar and consistent with the results of our study, other researchers have also reported the effect of CES on reducing craving. Sadeghi Bimorgh et al. did not report a significant difference between patients treated with 7 sessions of CES and patients in the placebo group in relapse, but regarding to our study, there was a significant difference in the level of depression, anxiety and stress between the intervention and placebo groups [1]. In the study by Taremian et al. 60 participants with opium use disorder were randomly divided into 3 groups, including active TDCS with methadone maintenance treatment, placebo transcranial direct current stimulation with methadone maintenance treatment. The results showed a significant reduction in depression, anxiety and opium craving in the active TDCS group compared to the other two groups [37]. In another study, Sharifi-Farshad et al. evaluated 40 right-handed male users of crystal heroin in two groups receiving TDCS and a control group that used placebo stimulation. They showed that TDCS significantly reduced drug craving in crystal heroin users [38].

Ideas that can be considered in future studies could include performing the CES interventions on female addicted people, increasing the total time of the intervention (it may enhance or prolong the therapeutic effects), longer follow-up of patients (to evaluate the long-term effectiveness of this method). Additionally, evaluation of the depression, anxiety and craving of people with other addictions, including smoking, or investigating the effect of CES with other CES devices on people undergoing methadone maintenance treatment could be subjects for future evaluation.

Among the limitations of this study, we can point out the non-cooperation of the patients and unwillingness to participate in the study in case of increased intervention in the treatment, the limited time of the study to be presented to the university.

## Conclusion

The results of this study showed that the use CES for 8 weeks during 30-minute sessions has no effect on reducing anxiety and depression of male addicted people with methadone maintenance treatment, but it can reduce drug craving in male addicted people undergoing methadone maintenance treatment. The technique of CES can be a valuable adjuvant therapeutic strategy in the clinical environment, but it is suggested that different frequencies and treatment times should be investigated to confirm the results of the present study.

In the present study the application of CES was on the male population, therefore, to ensure the generalization of the results to the entire general population (women and men), it is necessary to conduct a study with gender homogeneity and bigger sample size.

## Electronic supplementary material

Below is the link to the electronic supplementary material.

Supplementary Material 1

## Data Availability

The datasets used and/or analyzed during the current study available from the corresponding author on reasonable request.

## Abbreviations

WHO: World Health Organization
DBS: Deep brain stimulation
TMS: Transcranial magnetic stimulation
tDCS: Transcranial direct current stimulation
CES: Cranial electrotherapy stimulation
SPSS: Statistical Product and Service Solutions
